# One-size-fits-all or patient-tailored hemodynamic targets in post-cardiac arrest patients: an observational near-infrared spectroscopy study on cerebral autoregulation

**DOI:** 10.1186/cc14512

**Published:** 2015-03-16

**Authors:** C Genbrugge, K Ameloot, I Meex, W Boer, F Jans, W Mullens, M Dupont, B Ferdinande, J Dens, C Dedeyne

**Affiliations:** 1 ZOL Genk, Belgium

## Introduction

A subgroup of post-CA patients with disturbed cerebral autoregulation might benefit from higher mean arterial pressures (MAPs). We aimed to (1) investigate whether patients with disturbed autoregulation have a worse prognosis, (2) phenotype these patients, (3) define an individual optimal MAP and (4) investigate whether time under this individual optimal MAP is associated with outcome.

## Methods

A prospective observational study in 51 post-CA patients monitored with near-infrared spectroscopy.

## Results

(1) In multivariate analysis, patients with preserved autoregulation (33.65%) had a significant higher 180-day survival rate (OR = 4.62, 95% CI (1.06; 20.06), *P *= 0.04). (2) Phenotypically, a higher proportion of patients with disturbed autoregulation had pre-CA hypertension (31 ± 47 vs. 65 ± 49%, *P *= 0.02) suggesting that right shifting of autoregulation is caused by chronic adaptation of cerebral blood flow to higher blood pressures. Based on an index of autoregulation (COX), the average COX-predicted optimal MAP was 85 mmHg in patients with preserved and 100 mmHg in patients with disturbed autoregulation. (3) An individual optimal MAP could be determined in 33/51 patients. (4) The time under the individual optimal MAP was negatively associated with survival (OR = 0.97, 95% CI (0.96; 0.99), *P *= 0.02). The time under previously proposed fixed targets (65, 70, 75, 80 mmHg) was not associated with a differential survival rate.

## Conclusion

Cerebral autoregulation was shown to be disturbed in 35% of post-CA patients of which a majority had pre-CA hypertension. Disturbed cerebral autoregulation within the first 24 hours after CA is associated with a worse outcome. In contrast to uniform MAP goals, the time spent under a patient-tailored optimal MAP, based on an index of autoregulation, was negatively associated with survival.

